# P-1875. A Quality Initiative to Understand Risk Factors for Pneumonia Readmissions at a Regional Medical Center

**DOI:** 10.1093/ofid/ofae631.2036

**Published:** 2025-01-29

**Authors:** Chloe A Morgan, Jaime Jaronko, Frank Schembri, Kamran Manzoor

**Affiliations:** Tufts University School of Medicine, Melrose, Massachusetts; South Shore Health, Boston, Massachusetts; South Shore Health, Boston, Massachusetts; Tufts University School of Medicine, South Shore Health, Boston, Massachusetts

## Abstract

**Background:**

Pneumonia is a significant cause of hospitalizations in the United States, with 1 million pneumonia-related hospital admissions in 2014. Additionally, patients discharged after pneumonia treatment have a high all-cause 30-day readmission rate between 17%-25%. In order to create a quality improvement initiative to decrease pneumonia readmissions, we sought to identify risk factors, rates, and causes of readmission in patients with index admissions due to pneumonia.

**Figure:**
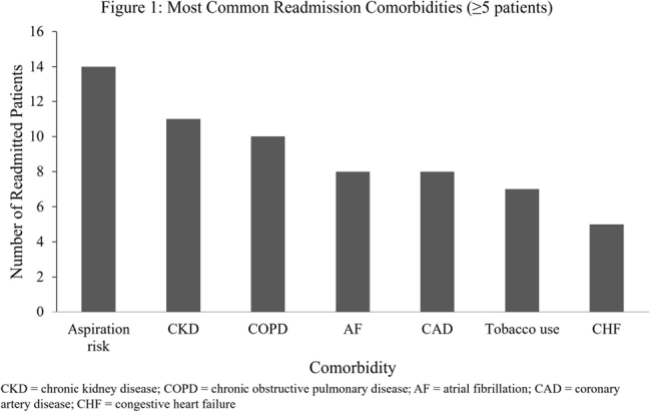

Most Common Readmission Comorbidities

**Methods:**

We performed a manual review of deidentified electronic medical record (EMR) data to select adult patients hospitalized for pneumonia for four consecutive months over two years (August to November 2022 and 2023) at a regional medical center. We assessed the primary cause of readmission for patients readmitted within 30 days of discharge to understand the risk factors, associated comorbidities, and care received.

**Results:**

EMR review identified 161 hospitalizations due to pneumonia in 8 months. Of these, 24 patients (15%) were readmitted within 30 days of hospital discharge, and most (15) of these readmissions were within 15 days. Notably, only 2 readmissions (8.3%) were due to pneumonia-related causes. The two most common causes for readmission were infections other than pneumonia (25%) and renal failure (21%). Most readmissions (14 readmissions [58%]) were classified as at risk for aspiration, and 4 (17%) were associated with opioid use. The most frequently seen comorbidities in readmission were aspiration risk/dysphagia, chronic kidney disease, chronic obstructive pulmonary disease, and atrial fibrillation (Figure 1). 15 out of 24 patients (63%) did not have a 30-day follow-up with their primary care provider.

**Conclusion:**

Our data suggest that most readmissions were not directly related to pneumonia, further suggesting that patient comorbidities and risk factors play a more significant role. Quality improvement programs should focus on a broad, multidisciplinary approach to identify the most vulnerable patient populations and associated risk factors and prioritize prompt outpatient follow-up with a primary care physician.

**Disclosures:**

All Authors: No reported disclosures

